# Supplementary material for: Nutritional Therapy Modulates Intestinal Microbiota and Reduces Serum Levels of Total and Free Indoxyl Sulfate and P-Cresyl Sulfate in Chronic Kidney Disease (Medika Study)
Source: J Clin Med. 2019 Sep 10;8(9):1424. doi: 10.3390/jcm8091424 (PMC6780815; doi:10.3390/jcm8091424)
Supplement: Supplementary file 1 [file jcm-08-01424-s001.pdf]

Supplemental Material

MEDIKA STUDY

Table of Contents

Study Design, Risk analysis, possible problems and solutions …..............................pag. 3

Supplementary Figures ....pag. 5

REFERENCES …………………………………………………………………………pag. 7

MATERIALS AND METHODS

Study Design

As previously described[1], 60 incident patients referred to a tertiary nephrology clinic were recruited on the basis of the following inclusion criteria: (i) age > 18 years; (ii) CKD stages 3B–4 [estimated glomerular filtration rate (eGFR) between 15 and 45mL/min/1.73m2, glomerular filtration rate calculated with creatinine-EPI]; (iii) adherence to prescribed therapies. The exclusion criteria were: (i) change in the eGFR by >30% within the last 3 months; (ii) previously (<3 months) acute intercurrent illness; (iii) severe undernutrition on the basis of <20 kg/m2 body mass index (BMI) and serum albumin level less than 3.2 g/dL, or BMI <17.5 kg/m2 independent of albumin values, or a body weight reduction of >5% within the last month or >10% within the last 6 months; (iv) breastfeeding or pregnancy; (v) chronic treatment with steroids or cytotoxic drugs; (vi) fast progressing glomerulonephritis; (vii) active systemic lupus erythematosus (SLE) and vasculitis; (viii) gastrointestinal diseases (Crohn's disease, ulcerative colitis, coeliac sprue, stypsis); (ix) infectious diseases; (x) cardiac failure New York Heart Association (NYHA) stages III–IV; (xi) advanced liver cirrhosis; (xii) active cancer; (xiii) severe encephalopathy associated with lack of spontaneous feeding; (xiv) oxygen treated chronic obstructive respiratory diseases; (xv) use of antibiotics and/or probiotics until 30 days before the start of the study; and (xvi) psychiatric illness or inability to assess at follow-up.

Risk analysis, possible problems and solutions

Since 1963, studies have confirmed that, in order to maintain a positive nitrogen balance, an adequate energy intake (30-35 Kcal/kg/day) must be provided, avoiding so the risk that the reduced protein intake may result in malnutrition. The ability of the human body to

synthesize new proteins, even in conditions of very low protein intake (0.3 g/kg/day) is referred to as positive nitrogen balance.

An amount of calories of not less than 30 Kcal/kg and a dietary protein content of not less than 0.3 g/kg are reported to prevent the protein-calorie malnutrition. It is necessary to prevent the onset of such issue, by controlling the patient's body weight variation, and by collecting laboratory data which are indicative of caloric-protein malnutrition[2].

Supplemental figure 1. Study Design. Software randomization divided CKD patients into two treatment arms (A and B), with a 1:1 ratio, each including 30 patients alternatively undergoing 3 dietary regimens as following: Arm A - 3 months of FD, 6 months of VLPD, 3 months of FD and 6 months of MD; Arm B - 3 months of FD, 6 months of MD, 3 months of FD and 6 months of VLPD.

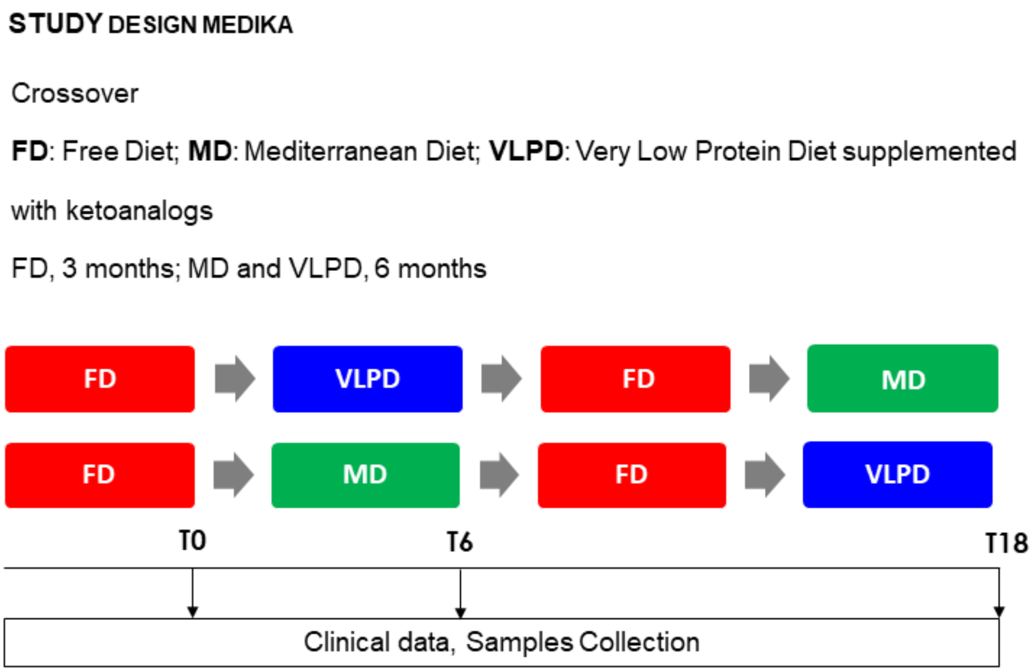

Supplemental Figure 2. Correlation (of the serum levels of azotemia, total indoxyl sulfate (IS) (Fig 2A), and total p-cresyl sulfate (PCS) (Fig 2B) in FD, MD and VLPD

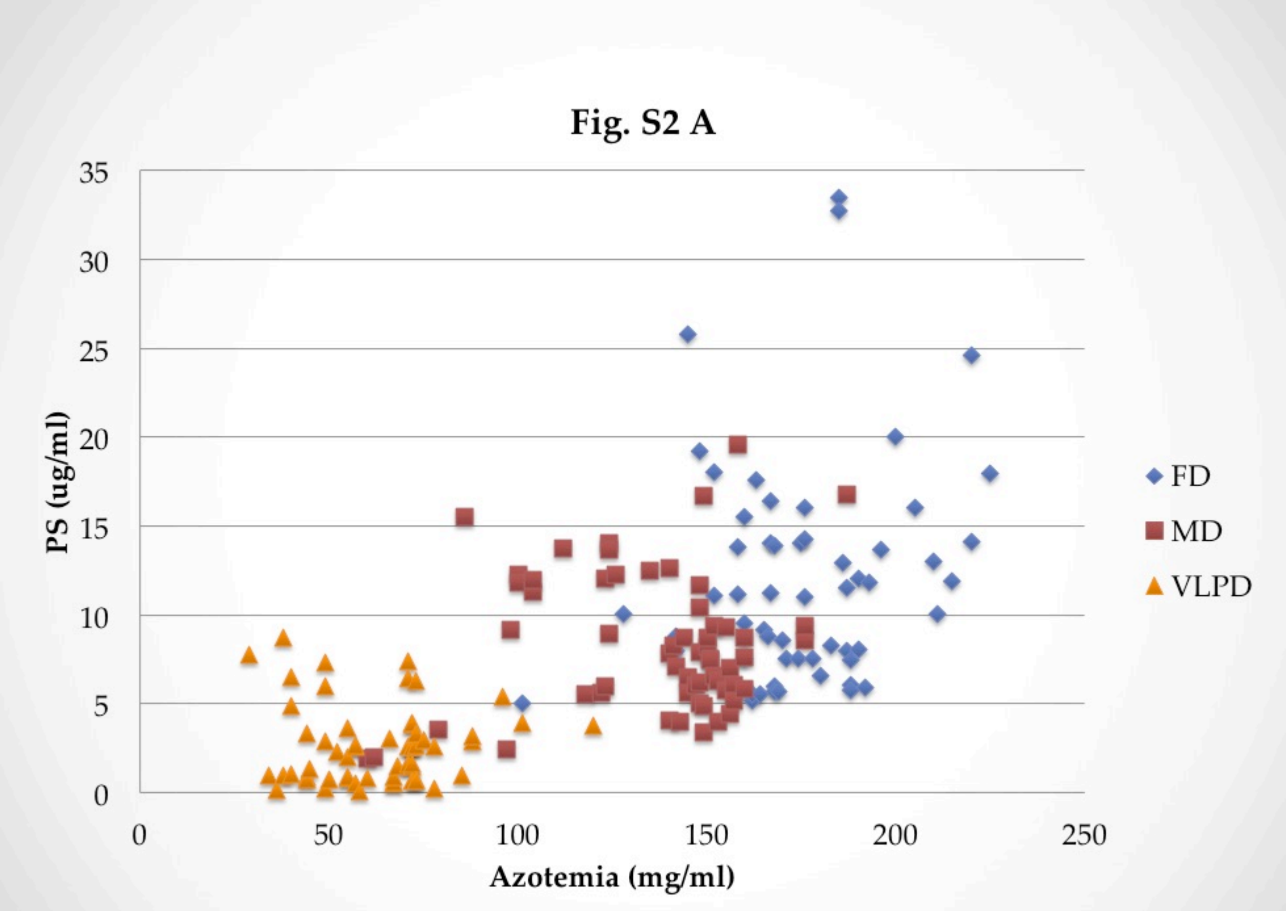

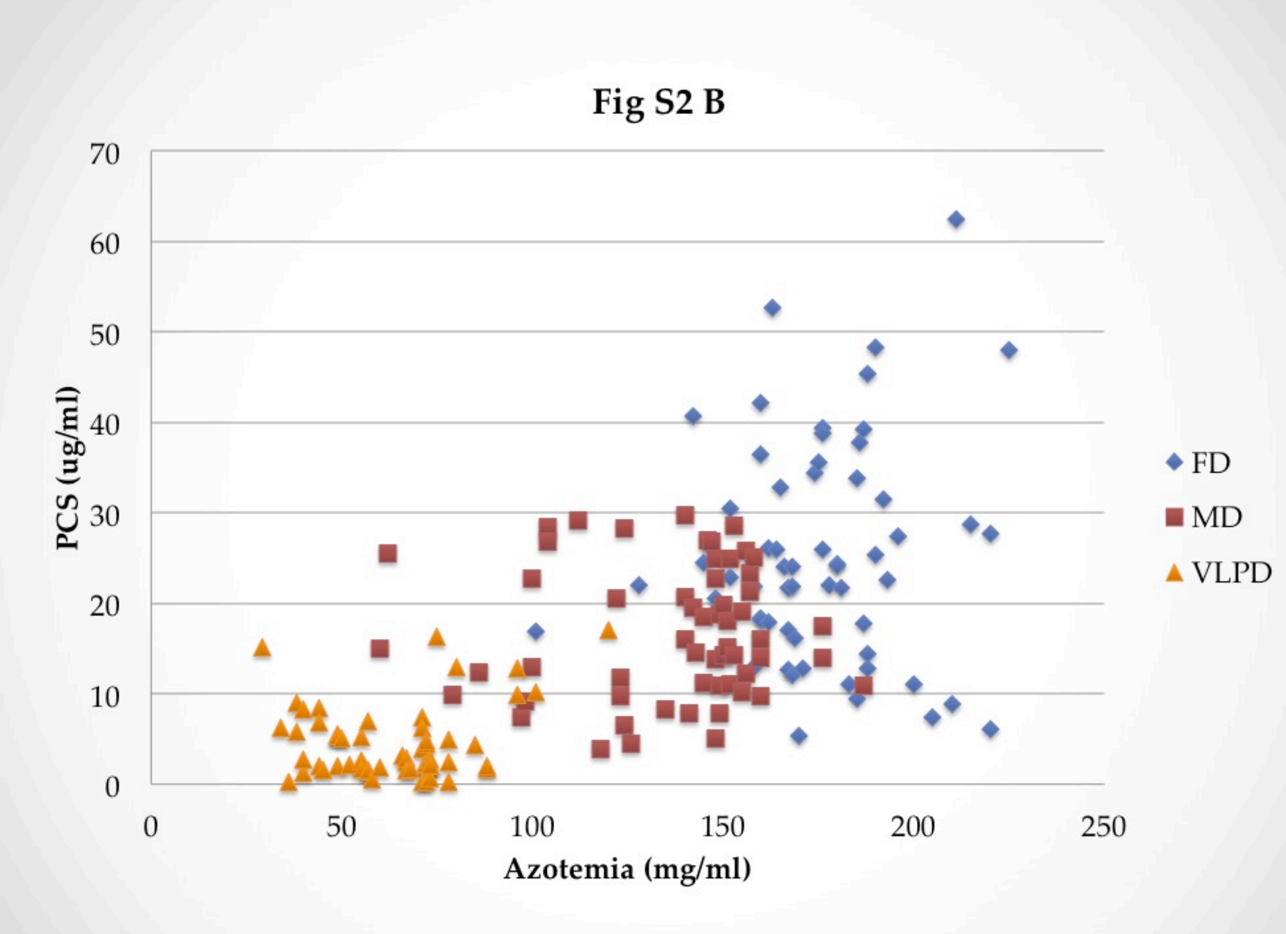

Fig S2 B
PCS (ug/ml)
70
60
50
40
30
20
10
0
0
50
100
150
200
250
Azotemia (mg/ml)
FD
MD
VLPD

REFERENCES

1. Di Iorio BR, Marzocco S, Bellasi A, et al. Nutritional therapy reduces protein carbamylation through urea lowering in chronic kidney disease. Nephrol Dial Transplant. 2017:1–10. doi: 10.1093/ndt/gfx203.
2. Windahl K, Faxén Irving G, Almquist T, et al. Prevalence and Risk of Protein-Energy Wasting Assessed by Subjective Global Assessment in Older Adults With Advanced Chronic Kidney Disease: Results From the EQUAL Study. J Ren Nutr. 2018;28(3):165-174. doi: 10.1053/j.jrn.2017.11.002.
